# Reactive Oxygen Species as Mediators of Gametophyte Development and Double Fertilization in Flowering Plants

**DOI:** 10.3389/fpls.2020.01199

**Published:** 2020-08-05

**Authors:** Subramanian Sankaranarayanan, Yan Ju, Sharon A. Kessler

**Affiliations:** ^1^ Department of Botany and Plant Pathology, Purdue University, West Lafayette, IN, United States; ^2^ Purdue Center for Plant Biology, Purdue University, West Lafayette, IN, United States

**Keywords:** reactive oxygen species (ROS), plants, reproduction, development, pollination, fertilization, gametophyte

## Abstract

Reactive oxygen species (ROS) are toxic by-products of aerobic metabolism. In plants, they also function as important signaling molecules that regulate biotic and abiotic stress responses as well as plant growth and development. Recent studies have implicated ROS in various aspects of plant reproduction. In male gametophytes, ROS are associated with germline development as well as the developmentally associated programmed cell death of tapetal cells necessary for microspore development. ROS have a role in regulation of female gametophyte patterning and maintenance of embryo sac polarity. During pollination, ROS play roles in the generation of self-incompatibility response during pollen-pistil interaction, pollen tube growth, pollen tube burst for sperm release and fertilization. In this mini review, we provide an overview of ROS production and signaling in the context of plant reproductive development, from female and male gametophyte development to fertilization.

## Introduction

**Figure 1:**
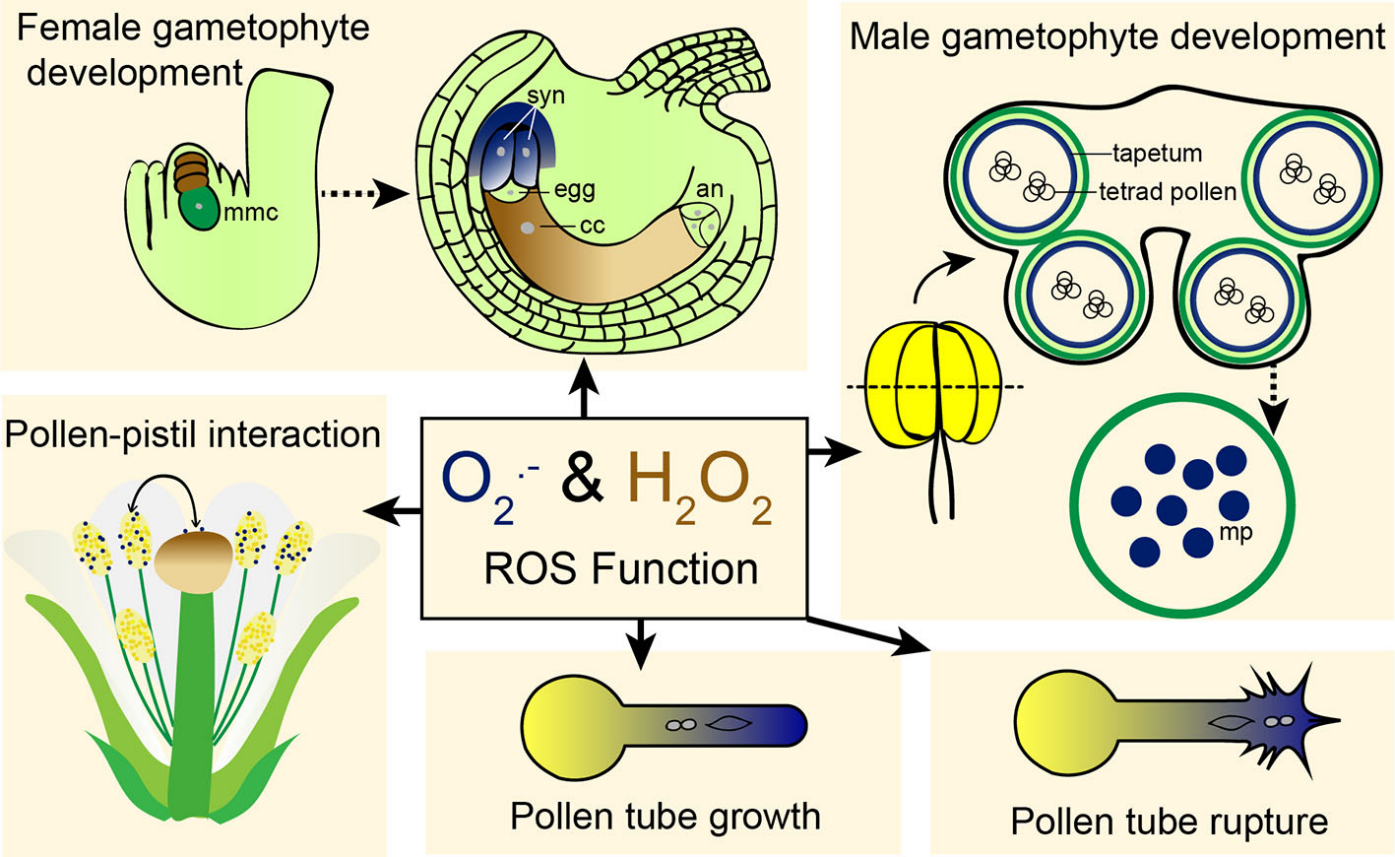
ROS is involved in different aspects of plant reproduction. ROS has been shown to regulate female and male gametophyte development, pollen-pistil interaction, pollen tube growth, and pollen tube rupture. Dark blue color indicates superoxide (the same color as NBT staining used for superoxide detection) and brown indicates hydrogen peroxide (the same color as DAB staining used for detection of hydrogen peroxide). mmc, megaspore mother cell; syn, synergid cells; cc, central cell; an, antipodal cells; mp, mature pollen.

Reactive oxygen species (ROS; e.g., O_2_^.−^, H_2_O_2_, OH., and ^1^O_2_) are constantly generated in various cellular compartments as by-products of aerobic metabolism (Mittler, 2017). Major sources of ROS generation in plant cells include mitochondrial respiration, photosynthesis in chloroplast, photorespiratory reactions in peroxisomes, and NADPH oxidases localized to the apoplast (Mhamdi and Van Breusegem, 2018). Each ROS species is unique with a distinct half-life and biochemical reactivity. For example, singlet oxygen (^1^O_2_) and superoxide (O_2_^.−^) both have a half-life (t_1/2_) of 1 to 4 μs, but have different modes of action. ^1^O_2_ oxidizes lipids, proteins, and guanine residues of DNA; while O_2_^.−^ reacts with Fe-S proteins. Hydroxyl radicals (OH^.^) are the most unstable ROS with a t_1/2_ of 1 ns and react with all biomolecules in a cell including DNA, RNA, lipids, and proteins. Hydrogen peroxide (H_2_O_2_) is more stable with a t_1/2_ of 1 ms and hence involved in cellular signaling (Mittler, 2017). While ROS have the potential to be toxic and must be neutralized to prevent damage to cellular components, plants have also evolved mechanisms to utilize ROS for their development (Mhamdi and Van Breusegem, 2018). In order to use ROS as signaling molecules, their levels are balanced between production and breakdown *via* complex redox networks comprised of several antioxidant enzymes and non-enzymatic reactions (Mittler, 2017). Catalases, peroxidases, ascorbate peroxidases, glutathione peroxidases, and peroxiredoxins are all involved in H_2_O_2_ metabolism (Mittler, 2017). O_2_^.−^ is detoxified by the enzyme superoxide dismutases (SODs) and other molecules like flavonoids and ascorbate (Mittler, 2017). OH^.^ are detoxified by molecules like flavonoids, proline, sugars, and ascorbate; while carotenoids and α-tocopherol are involved in detoxification of ^1^O_2_ (Mittler, 2017). ROS function as important signaling molecules in plants through their crosstalk with phytohormones and other pathways modulate plant growth and development, responses to biotic and abiotic stress, autophagy, and programmed cell death (PCD) (Considine and Foyer, 2014; Noctor et al., 2018; Huang et al., 2019). Here, we discuss the emerging evidence supporting roles for ROS in several stages of plant reproduction (**Figure 1**).

## Involvement of ROS in Male and Female Gametophyte Development

Gametophytes are multicellular haploid structures that contain the gametes. The male gametophyte is the pollen grain, a three-celled structure made up of the vegetative cell and two sperm cells. The female gametophyte, also known as the embryo sac, is a seven-celled structure consisting of the gametes (the egg and central cell), two synergid cells, and three antipodal cells. ROS homeostasis is essential for both male and female gametophyte development. Respiratory burst oxidase homologs (Rboh; NADPH oxidase) dependent O_2_^.−^ production occurs during both male and female gametophyte development (Jiménez-Quesada et al., 2016).

### ROS in Male Gametophyte Development

The tapetum is a layer of cells in the anther that provides nutrients for pollen development and materials for pollen wall formation and is, therefore, essential for male gametophyte development. Pollen mother cells or meiocytes are encased within a tapetum-derived callose wall and undergo meiosis to produce tetrads of haploid microspores (Gómez et al., 2015). Developmentally regulated PCD leads to tapetum degeneration that results in microspore release into the anther locule. Post-meiotic male gametophyte development then proceeds with two rounds of mitotic divisions to produce the vegetative cell and two sperm cells and subsequent pollen wall formation (Gómez et al., 2015). Thus, PCD of the tapetum is essential for proper pollen development. ROS have been shown to play a key role in tapetum function and death in model dicots and in rice. NADPH oxidases encoded by the gene family contribute to the production of ROS (Lamb and Dixon, 1997; Jiménez-Quesada et al., 2016). In Arabidopsis, *RbohE* is expressed in the anther tapetum during microspore development. Loss-of-function *rbohE* mutants had defective pollen development that was associated with reduced ROS levels and delayed tapetal degeneration (Xie et al., 2014). Conversely, overexpression of *RbohE* in tapetal cells led to increased ROS and precocious tapetal degeneration that also interfered with pollen development, indicating that precise regulation of ROS levels in the tapetum is essential for pollen development (Xie et al., 2014). Similarly, several *Rboh*s are preferentially expressed in tobacco and tomato anthers. Manipulation of ROS levels by treatment with the NADPH oxidase inhibitor diphenyleneiodonium chloride (DPI) during anther development in tomato and tobacco impaired both tapetal degeneration and pollen development (Yu et al., 2017).

While the Rboh proteins seem to have a direct influence on ROS generation in the tapetum, indirect regulators have also been implicated. For example, the homeobox transcription factor, Os*MADS3*, regulates ROS homeostasis during anther development in rice. Os*MADS3* is expressed in the tapetum and microscopes during late anther development. *Osmads3* mutant had increased accumulation of O_2_^.−^, defective anther walls and pollen sterility (Hu et al., 2011). OsAGO2, a member of ARGONAUTE (AGO) family, is involved in epigenetic regulation of anther development by modulating DNA methylation in the *Hexokinase* (*OsHXK*) promoter region to downregulate OsHXK expression (Zheng et al., 2019). Knockdown of *OsAGO2* led to the upregulation of *Rboh* gene expression, overaccumulation of ROS, and abnormal anther development with premature initiation of PCD and pollen abortion. Similarly, overexpression of OsHXK also led to increased ROS production, tapetal degeneration, and pollen abortion (Zheng et al., 2019).

Either too much or too little ROS can disrupt tapetum-regulation of pollen development, indicating that ROS may have a role in normal development through their participation in carefully balanced redox reactions. Class III peroxidases constitute one of the major redox gene regulation networks in plants (Oliveira et al., 2019). The class III peroxidases can function as catalytic enzymes that consume hydrogen peroxide to oxidize phenolic compounds and/or generate ROS. Peroxidases have been implicated in diverse aspects of plant development and response to the environment, often involving cell wall modifications (Shigeto and Tsutsumi, 2016). In Arabidopsis, two class III peroxidase-encoding genes, *PEROXIDASE9* (*PRX9*) and *PRX40* are required for maintaining tapetum and microspore cell wall integrity during anther development in Arabidopsis (Jacobowitz et al., 2019). PRX9 and PRX40 were confirmed to be H_2_O_2_-dependent peroxidases that are capable of crosslinking extensins in the cell wall. *prx9prx40* double mutants displayed tapetum hypertrophy and pollen degeneration consistent with a lack of extensin cross-linking and compromised cell wall integrity (Jacobowitz et al., 2019). There are several members of class III peroxidases which are expressed in pollen (**Table 1**), suggesting a possible role for this gene family in pollen development by modulation of ROS levels. Taken together, it can be concluded that ROS dynamics regulates various aspects of male gametophyte development, including microspore development, pollen maturation, and tapetal degradation.

**Table 1:**
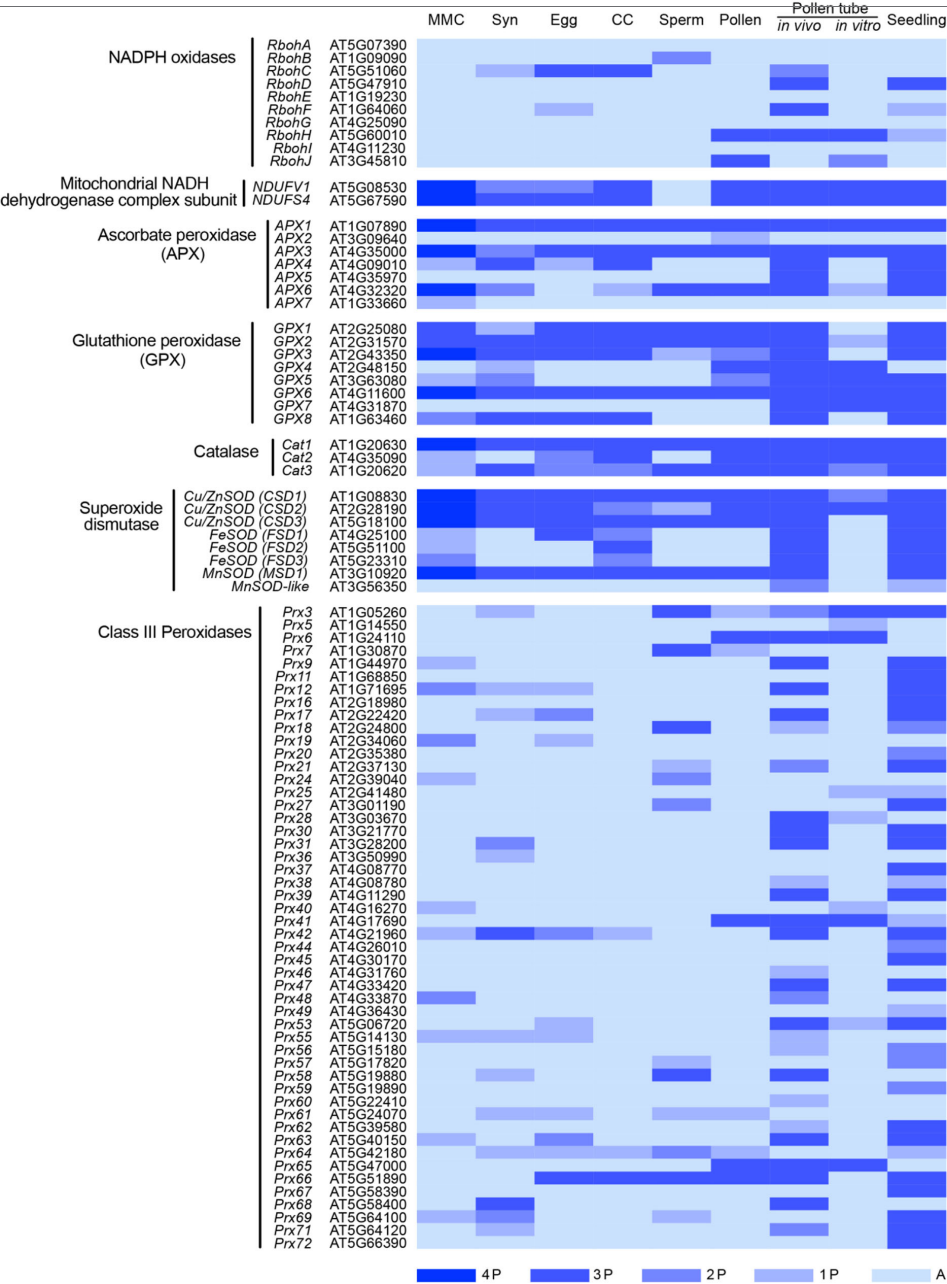
Annotation and expression of ROS production and scavenging genes in Arabidopsis reproductive cells.

The darkness of blue color indicates the likelihood of gene expression based on the number of experimental replicates with a present (P) call. 4 P, presence in four replicates; 3 P, presence in three replicates; 2 P, presence in two replicates; 1 P, presence in one replicate; A, absence of expression; MMC, megaspore mother cell; Syn, synergid; CC, central cell. Note that the total replicates for MMC are 4, and the total replicates for the rest cell types are 3. These data were compiled using the gene expression data obtained from Schmidt et al., 2011 (transcriptome), Wuest et al., 2010 (transcriptome), and Lin et al., 2014 (translatome).

### ROS in Female Gametophyte Development

Most angiosperms have polygonum-type female gametophytes composed of seven different cells: the egg cell, the central cell, two synergid cells, and three antipodal cells (Yadegari and Drews, 2004). ROS levels are tightly regulated during the process of megagametogenesis. In mature female gametophytes, superoxide and peroxide are detected in the central cell and absent from the antipodal cells (Martin et al., 2013a; Martin et al., 2013b). ROS generated by the mitochondria have an important role in regulation of cell fate and embryo sac polarity (Martin et al., 2013a; Martin et al., 2013b). Crucial roles of mitochondrial ROS during embryo sac development were demonstrated using *oiwa*, a female gametophytic mutant impaired in mitochondrial manganese-superoxide dismutase (MSD1) (Martin et al., 2013a; Martin et al., 2013b). In *oiwa*, high levels of ROS accumulate in the central cell as well as the micropylar cells. The high levels of peroxide and mitochondrial superoxide in *oiwa* mutants were correlated with a range of female gametophyte phenotypes, including mitotic arrest during megagametogenesis and mis-specification of egg apparatus cells leading to partial central cell identity (Martin et al., 2013a; Martin et al., 2013b).

A second link to mitochondrial ROS homeostasis and gametophyte development was provided by *athemn1*, a mutant defective in tetrapyrrole biosynthesis. *athemn1* mutant had increased ROS accumulation in developing anthers and embryo sacs (Pratibha et al., 2017). *athemn1* mutants displayed defects in gametophyte development, including nonviable pollen and embryo sacs with unfused polar nuclei. Central cell differentiation was also impaired in this mutant resulting in a defective endosperm development and an embryo developmental arrest (Pratibha et al., 2017). Transcriptomic data have revealed the presence of several genes involved in ROS regulation in the earliest stage of Female gametophyte (FG) development, the megaspore mother cell (MMC), and also in different cell types of mature female gametophyte (**Table 1**). For example, the main generators of mitochondrial ROS, *NDUFV1* and *NDUFS4* (NADH dehydrogenase subunits) (Zeng et al., 2017), are expressed in the MMC as well as in the cells of mature female gametophyte (**Table 1**). However, the roles of these genes in female gametophyte development is yet to be uncovered. Nevertheless, the research reviewed here strongly supports a role for ROS in female gametophyte development.

## ROS as Mediator of Pollen-Pistil Interactions

Successful pollination depends on a series of pollen-pistil interactions that initiate after compatible pollen lands on a receptive stigma. Pollen recognition, adhesion, hydration, germination, foot formation, and pollen tube growth need to occur before the pollen tube can be guided to the ovule for sperm delivery to the female gametes. Stigmas of several angiosperms accumulate ROS (mainly H_2_O_2_) constitutively (McInnis et al., 2006; Zafra et al., 2016). Stigmatic ROS is thought to be involved in signaling networks that promote pollen germination and pollen tube growth on the stigma (Lan et al., 2017). In ornamental kale (*Brassica oleracea* var. *acephala*), ROS-scavenging flavanoids and ROS play antagonistic roles in mediating pollination. Flavonoid levels decline as the stigma reaches maturity resulting in ROS accumulation in the stigma and allowing compatible pollination to occur (Lan et al., 2017). ROS has also been implicated in Self-Incompatibility (SI), the reproductive barrier that plants utilize to prevent self-pollination to promote genetic variability (Sankaranarayanan et al., 2015). Gametophytic SI in poppy (*Papaver rhoeas* L.), pear (*Pyrus pyrifolia* L.), and olive (*Olea europaea* L.) results from PCD of the self-pollen triggered by an increase in ROS levels inside the pollen tubes (Bosch and Franklin-Tong, 2008; Wilkins et al., 2011; Jiang et al., 2014; Serrano et al., 2015).

### ROS in Male Gametophyte Function

Tight regulation of ROS homeostasis is necessary for both pollen germination and tip growth. For example, ROS generation in the pollen is correlated with the initial event of pollen hydration on the stigma (Gao et al., 2016). KINB**γ** is a subunit of SNF1-related protein kinase 1 complex involved in the biogenesis of mitochondria and peroxisomes in Arabidopsis pollen. *kinβ*γ** mutant pollen accumulates less ROS than wild-type pollen and is compromised in its ability to hydrate and germinate on the stigma (Gao et al., 2016). ROS has also been shown to play a role in pollen germination in kiwifruit and blue spruce (Speranza et al., 2012; Maksimov et al., 2018).

ROS has also been implicated in tip growth of pollen tubes through the reproductive tract. Pollen tube growth is largely dependent on the distribution of ions through the plasma membrane as well as free inorganic ions like Ca^2+^,K^+^, Cl^-^, H^+^ in the cytoplasm termed “ion zoning” (Podolyan et al., 2019). Recent studies have revealed that H_2_O_2_ can control membrane potential in pollen tubes by regulating Ca^2+^ sensitive channels and ion transport during tip growth (Breygina et al., 2016; Maksimov et al., 2016).

NADPH dehydrogenases and NADPH oxidases are key enzymes for ROS generation in plant cells. ROS accumulates at the subapical and apical regions of growing pollen tubes where mitochondria are most abundant (Cárdenas et al., 2006). Inhibition of either NADPH dehydrogenases or NADPH-oxidases with DPI or NADPH oxidases-specific antisense oligodeoxynucleotides resulted in an inhibition of pollen tube growth (Cárdenas et al., 2006; Potocký et al., 2007). RbohH and RbohJ are NADPH oxidases that have Ca^2+^-induced ROS-producing activity and localized to the plasma membrane of the pollen tube tip (Kaya et al., 2014). The *rbohH rbohJ* double mutant pollen tubes exhibit high frequency growth oscillations correlated with growth-dependent Ca^2+^ bursts and increase in the rate of cell-wall exocytosis (Lassig et al., 2014). The double mutant is also defective in pollen tip growth and show reduced fertility as a consequence of defective ROS accumulation in the pollen tube cell wall (Kaya et al., 2014; Lassig et al., 2014; Kaya et al., 2015). Other Rboh family members, including *RbohC*, *RbohD*, *RbohF* are expressed in pollen tubes growing through the female tissues, suggesting their potential involvement in ROS generation in pollen tubes (**Table 1**). Rboh-induced ROS production was proposed to activate Ca^2+^ channels such as the cyclic nucleotide gated channels (CNGC) in the pollen tube (Wudick and Feijó, 2014). Interestingly, the CNGC family functions in pollen tube growth and *cngc7,8* double mutant has a similar pollen tube bursting and sterility phenotype as observed in the case of *rbohH rbohJ* double mutant (Tunc-Ozdemir et al., 2013). Further experiments are required to test this hypothesis and uncover any link between ROS and the CNGC in the pollen tube.

Receptor like kinases (RLKs) localized at the pollen tube tip are involved in maintaining cell wall properties of growing pollen tubes. Among the RLKs, ANXUR1 and 2 (ANX1 and 2) which are members of *Catharanthus roseus* RLK-1–like subfamily (CrRLK1L) coordinate cell wall integrity through NADPH oxidase-mediated ROS production (Boisson-Dernier et al., 2013). *anx1anx2* double mutants are sterile because pollen tubes rupture prematurely, preventing them from growing to fertilize the female gametophyte. A similar phenotype was observed in *rbohH* *rbohJ* double mutants. Over-expression of both ANX1-YFP and GFP-RbohH triggered over-accumulation of membrane and cell wall materials. Further experiments revealed that the NADPH oxidases function downstream of the ANX RLKs in the pollen tube integrity pathway (Boisson-Dernier et al., 2013). A recent study revealed that ANX1/2 interact with related RLKs BUDDHA’S PAPER SEAL1 and 2 (BUPS 1/2) and LORELEI-like GPI-anchored proteins 2 and 3 (LLG2/3) to form a receptor complex in the pollen tube. This receptor complex interacts with RAPID ALKALIZATION FACTORs 4 and 9 (RALF4/9) to regulate tip integrity and has also been linked to ROS production for pollen tube growth (Feng et al., 2019).

Maintenance of ROS homeostasis in pollen tubes is dependent on the availability of precursors and the abundance of antioxidant scavengers. An ABC transporter, ABCG28 was shown to be required for the apical accumulation of ROS in growing pollen tubes (Do et al., 2019). ABCG28 is involved in accumulation of secretory vesicles containing polyamines, precursors of ROS, at the growing tip of pollen tubes. *abcg28* mutant pollen tubes have altered hydrogen peroxide distribution and fail to localize polyamine to the growing tip, resulting in defective pollen tube growth (Do et al., 2019). Flavanols and anthocyanins are secondary metabolites that function as ROS scavengers in plants. The *anthocyanin reduced* (*are*) tomato mutant has reduced flavanol accumulation in pollen grains and tubes. In consonance, *are* mutant displayed elevated levels of ROS in pollen grains and impaired pollen viability, germination, tube growth, and tube integrity, resulting in reduced seed set (Muhlemann et al., 2018). ROS levels increase in pollen tubes in response to heat stress, thus flavanols are particularly important for protecting pollen tubes from the damaging effects of high ROS levels (Muhlemann et al., 2018). In pollen tubes, ROS functions a double edge sword in regulating cell wall integrity and tip growth. ROS levels must be tightly regulated in the pollen tubes to prevent it from reaching inhibitory levels.

### ROS in Female Gametophyte Function

After crossing the stigmatic barrier, the pollen tube grows through the style and transmitting tract, exits and navigates along the ovule funiculus, enters through the ovule micropyle, and bursts to release two sperm cells into the female gametophyte. Double fertilization occurs when one sperm fuses to the egg cell, and the other with the central cell (Johnson et al., 2019). The role of ROS in pollen tube growth through the pistil and during pollen tube reception has been reviewed in detail recently (Zhang et al., 2020). We will focus on ROS and female gametophyte function for the remainder of this review.

The major links between ROS and female gametophyte function come from studies of the synergid cells in Arabidopsis. The two synergids are accessory cells whose main function is to communicate with the pollen tube. Synergids secrete small cysteine-rich peptides LUREs to guide the pollen tube toward the ovule (Okuda et al., 2009; Higashiyama and Takeuchi, 2015). Receptors localized to the pollen tube tip perceive these LURE peptides secreted from the synergid cells to guide it toward the ovules (Takeuchi and Higashiyama, 2016; Wang et al., 2016). After being attracted to the female gametophyte, pollen tubes pause outside the synergids, near the filiform apparatus, a membrane-rich region that contains important signaling proteins, so that signals from the synergid can be perceived and translated to changes in the pollen tube tip that will allow the pollen tube to burst and deliver the sperm cells to the female gametes (Kessler and Grossniklaus, 2011). This process is known as pollen tube reception.

The female gametophyte has been proposed to prepare for pollen tube arrival by creating an oxidative environment required for pollen tube reception at the synergids (Martin et al., 2013a). Evidence for this hypothesis comes from ROS staining experiments. Hydrogen peroxide was detected in synergid cells after pollen was applied to the stigma, but before pollen tubes reached the ovules, suggesting that pollination triggers an oxidative burst in the embryo sac (Martin et al., 2013a). In addition, staining with H_2_DCF-DA, a general ROS stain, revealed high ROS levels in the ovule micropyle, at or near the synergid filiform apparatus (Duan et al., 2014). Pistil feeding experiments with ROS scavengers and inhibitors led to defects in pollen tube reception, with the pollen tubes attracted normally to ovules but continuing to grow and failing to burst and release the sperm for double fertilization (Duan et al., 2014). This pollen tube overgrowth phenotype is very similar to that seen in mutants of synergid-expressed genes that are necessary for communication between the female and male gametophytes. Three synergid-expressed CrRLK1L genes have been implicated in pollen tube reception: FERONIA (FER), HERKULES1 (HERK1), and ANJEA (ANJ) (Galindo-trigo et al., 2020). As discussed previously in this review, the pollen tube-expressed CrRLK1L genes *ANX1/2* have been directly linked to Rboh (Boisson-Dernier et al., 2013). FER has also been linked to Rho of Plants (ROP) protein-signaling and RboH-mediated ROS production in root hairs (Duan et al., 2010). *fer* mutants have pollen tube overgrowth and do not accumulate ROS in the micropyles of ovules, consistent with the inhibitor feeding experiments and suggesting that micropylar ROS is an important component of normal pollen tube reception. However, *herk1 anj* double mutants have pollen tube overgrowth but still accumulate micropylar ROS (Galindo-trigo et al., 2019), indicating that the link between micropylar ROS and synergid control of pollen tube behavior may be more complex than previously thought. Higher resolution imaging of ROS dynamics during pollen tube arrival to the synergid cells in *fer*, *lre*, *herk1*, and *anj* mutants and a better understanding of the effects of ROS on pollen tube bursting are essential to further clarify the role of ROS during pollen tube reception.

The synergid-expressed mildew resistance locus o (MLO) gene *NORTIA* (*NTA*, also known as *MLO7*) also participates in pollen tube reception, with *nta* mutants displaying pollen tube overgrowth (Kessler et al., 2010). Other members of the *MLO* gene family play a role in powdery mildew infection and have been proposed to negatively regulate localized ROS production at powdery mildew penetration sites and to play a role in modulating the threshold for ROS-induced cell death (Cui et al., 2018). The biochemical function of NTA is not known, but it accumulates in the Golgi during synergid differentiation and then at the filiform apparatus during pollen tube reception (Jones et al., 2017), consistent with a possible role in regulating the synergid response to extracellular ROS in the micropyle. MLOs could also be indirectly linked to ROS during pollen tube reception. A recent study reported that pollen tube-expressed MLOs regulate pollen tube growth direction by recruiting and interacting with the calcium channel CNGC18 to the plasma membrane in order to modify Ca^2+^ gradients in the pollen tube (Ju and Kessler, 2020; Meng et al., 2020). NTA regulates Ca^2+^ oscillations in synergids during pollen tube reception (Ngo et al., 2014). Micropylar ROS and NTA could both play roles in regulating calcium flux at the filiform apparatus.

The final stage of pollen tube reception is pollen tube bursting to release the sperm cells. Pollen tube discharge also completes the process of synergid degeneration that is initiated by interaction of pollen tube with the synergid (Leydon et al., 2015). Synergid degeneration occurs as a PCD response (Li et al., 2009) and ROS is known to drive PCD responses in plants cells (Van Breusegem and Dat, 2006). ROS could have a role in initiation of synergid cell degeneration by initiating a PCD response, though direct evidence is still lacking. In animals, ROS and NO have been implicated in sperm activation, acquisition of hyperactivated motility, acrosome reaction, egg activation and fertilization (Kuo et al., 2000; Ford, 2004; de Lamirande and O’Flaherty, 2008). However, ROS functions as a negative regulator of sperm-egg fusion by oxidation of sperm sulfhydryl proteins in mice (Mammoto et al., 1996). A role for ROS in sperm activation, motility and sperm-egg fusion has yet to be uncovered in plants.

## Conclusions and Perspectives

Research carried out over the past few decades has revealed critical roles for ROS in plant reproduction (**Figure 1**). ROS-producing and scavenging enzymes have been directly implicated in male gametophyte development and function. Up to this point, links between female gametophyte function and ROS accumulation have been based solely on detection of ROS by staining of wild-type and signaling mutant ovules, but no genetic evidence has been reported to directly link ROS to pollen tube reception and fertilization. Single cell transcriptase and translatome analysis of gametophytic cells revealed the expression of several genes involved in ROS production and scavenging, suggesting a tight regulation in synthesis and breakdown of ROS in these gametophytic **Table 1**; (Wuest et al., 2010; Schmidt et al., 2011; Lin et al., 2014). Reverse genetic analysis using T-DNA mutants of these genes will further shed light into the role ROS in plant reproduction.

Quantitative data on ROS levels in gametophytic cells has been limited due to the lack of tools and methodologies to quantify various ROS species in real time. The commonly used small molecule ROS detectors (DAB, NBT, H2DCFA, etc) are irreversible and only amenable to single timepoint quantifications and are often sensitive to environmental conditions such as pH, which can give misleading results (Erard et al., 2018). The application of genetically encoded and reversible ROS sensors, HyPer and roGFP2-Orp1 (Hernández-Barrera et al., 2013; Fichman et al., 2019; Nietzel et al., 2019), to detect intracellular ROS and the development of new sensors to detect extracellular ROS will enable real-time monitoring of ROS production and distribution during plant reproduction.

## Author Contributions

All authors listed have made substantial, direct, and intellectual contribution to the work and approved it for publication.

## Funding

This work is supported by Purdue University Start-up funds to SK.

## Conflict of Interest

The authors declare that the research was conducted in the absence of any commercial or financial relationships that could be construed as a potential conflict of interest.
